# Development and characterisation of a novel glucagon like peptide-1 receptor antibody

**DOI:** 10.1007/s00125-017-4491-0

**Published:** 2017-11-09

**Authors:** Emma K. Biggs, Lihuan Liang, Jacqueline Naylor, Shimona Madalli, Rachel Collier, Matthew P. Coghlan, David J. Baker, David C. Hornigold, Peter Ravn, Frank Reimann, Fiona M. Gribble

**Affiliations:** 1 Department of Cardiovascular and Metabolic Disease, MedImmune Ltd, Granta Park, Cambridge, UK; 2 Department of Antibody Discovery and Protein Engineering, MedImmune Ltd, Granta Park, Cambridge, CB21 6GH UK; 3 University of Cambridge Metabolic Research Laboratories, WT-MRC Institute of Metabolic Science, Addenbrooke’s Hospital, Hills Road, Cambridge, CB2 0QQ UK; 4 In Vivo Sciences – UK, AstraZeneca, The Babraham Institute, Cambridge, UK

**Keywords:** Antagonism, Antibody, GLP1R, Incretin, Phage display

## Abstract

**Aims/hypothesis:**

Glucagon like peptide-1 (GLP-1) enhances glucose-dependent insulin secretion by binding to GLP-1 receptors (GLP1Rs) on pancreatic beta cells. GLP-1 mimetics are used in the clinic for the treatment of type 2 diabetes, but despite their therapeutic success, several clinical effects of GLP-1 remain unexplained at a mechanistic level, particularly in extrapancreatic tissues. The aim of this study was to generate and characterise a monoclonal antagonistic antibody for the GLP1R for use in vivo.

**Methods:**

A naive phage display selection strategy was used to isolate single-chain variable fragments (ScFvs) that bound to GLP1R. The ScFv with the highest affinity, Glp1R0017, was converted into a human IgG1 and characterised further. In vitro antagonistic activity was assessed in a number of assays: a cAMP-based homogenous time-resolved fluorescence assay in GLP1R-overexpressing cell lines, a live cell cAMP imaging assay and an insulin secretion assay in INS-1 832/3 cells. Glp1R0017 was further tested in immunostaining of mouse pancreas, and the ability of Glp1R0017 to block GLP1R in vivo was assessed by both IPGTT and OGTT in C57/Bl6 mice.

**Results:**

Antibodies to GLP1R were selected from naive antibody phage display libraries. The monoclonal antibody Glp1R0017 antagonised mouse, human, rat, cynomolgus monkey and dog GLP1R. This antagonistic activity was specific to GLP1R; no antagonistic activity was found in cells overexpressing the glucose-dependent insulinotropic peptide receptor (GIPR), glucagon like peptide-2 receptor or glucagon receptor. GLP-1-stimulated cAMP and insulin secretion was attenuated in INS-1 832/3 cells by Glp1R0017 incubation. Immunostaining of mouse pancreas tissue with Glp1R0017 showed specific staining in the islets of Langerhans, which was absent in *Glp1r* knockout tissue. In vivo, Glp1R0017 reversed the glucose-lowering effect of liraglutide during IPGTTs, and reduced glucose tolerance by blocking endogenous GLP-1 action in OGTTs.

**Conclusions/interpretation:**

Glp1R0017 is a monoclonal antagonistic antibody to the GLP1R that binds to GLP1R on pancreatic beta cells and blocks the actions of GLP-1 in vivo. This antibody holds the potential to be used in investigating the physiological importance of GLP1R signalling in extrapancreatic tissues where cellular targets and signalling pathways activated by GLP-1 are poorly understood.

**Electronic supplementary material:**

The online version of this article (10.1007/s00125-017-4491-0) contains peer-reviewed but unedited supplementary material, which is available to authorised users.

## Introduction

The incretin hormone glucagon like peptide-1 (GLP-1) is secreted from enteroendocrine L cells following ingestion of food, and enhances glucose-dependent insulin secretion by activating GLP-1 receptors (GLP1R) on pancreatic beta cells [1]. GLP-1 mimetics are available in the clinic for the treatment of type 2 diabetes [2], but several effects of GLP-1 remain unexplained at a mechanistic level, particularly in extrapancreatic tissues.

When considering GLP1R expression, the field of research has been limited by a lack of specificity of commercially available GLP1R antibodies [3]. GLP1R is a G-protein-coupled receptor (GPCR); thus, generating antibodies is inherently difficult due to the seven transmembrane domains of the receptor. An alternative method for studying receptor expression has been transgenic expression of fluorescent reporters downstream of the *Glp1r* promoter [4]. Aside from this, the monoclonal antibody mAb3F52 with specificity for human and monkey GLP1R has been generated and used in immunostaining [5]. Combined, these methods have been used to report GLP1R expression across several tissues [4–6].

The seemingly widespread expression of GLP1R suggests that GLP-1 has a number of functions apart from enhancing glucose-induced insulin secretion. Within the pancreas, GLP-1 inhibits glucagon secretion from alpha cells, and stimulates somatostatin secretion from delta cells. Other proposed functions include stimulation of natriuresis in the kidneys, decrease of food intake via signalling in the central nervous system, modulation of heart rate, and cardioprotection in myocardial ischaemia [7]. Physiological effects of GLP-1 mimetics in the clinic include decreased cardiovascular risk and increased risk of retinopathy, although this varies between studies and may depend on the agonist used [8–10]. Uncertainty surrounds whether these additional effects are mediated directly via GLP1R on affected tissues, indirectly via GLP1R activation in neurons or through GLP1R-independent pathways [11].

GLP1R antagonists could be used to address some of these functional questions. The objective of this study was to generate and characterise a monoclonal antagonistic antibody for GLP1R that could be used to block GLP1R signalling in vivo. In comparison to the peptide antagonist exendin 9–39, an antibody would provide the advantage of having an extended half-life for use in subchronic functional studies. As off-target effects have also been observed for exendin 9–39 [12], another major advantage of a GLP1R antagonistic antibody is specificity for GLP1R. Here we developed an antagonistic antibody against GLP1R, and characterised it in a number of in vitro assays and in vivo studies using lean C57/Bl6 mice, which are well established for studying glucose homeostasis in the context of diabetes.

## Methods

### Compounds and solutions

Unless otherwise stated, chemicals were obtained from Sigma-Aldrich (Poole, UK). Peptides were purchased from Bachem (Bubendorf, Switzerland). The GLP1R extracellular domain (ECD) was produced in *Escherichia coli*, purified as previously described [13] and biotinylated using EZ-link Sulfo-NHS-LC-Biotin (Thermo Fisher Scientific, Loughborough, UK).

Saline solution for imaging experiments contained (mmol/l): 138 NaCl, 4.5 KCl, 4.2 NaHCO_3_, 1.2 NaH_2_PO_4_, 2.6 CaCl_2_, 1.2 MgCl_2_ and 10 HEPES (pH 7.4, NaOH). KRB contained (mmol/l): 120 NaCl, 3.5 KCl, 1.2 KH_2_PO_4_, 1.2 MgSO_4_, 2.5 CaCl_2_, 25 NaHCO_3_ (pH 7.2, NaOH). Assay buffer for calcium measurements and ligand-binding assays was Hanks’ balanced salt solution supplemented with 25 mmol/l HEPES and 0.1% (wt/vol.) BSA (pH 7.4); for the cAMP homogenous time-resolved fluorescence (HTRF) assay, the buffer was also supplemented with 0.5 mmol/l 3-isobutyl-1-methylxanthine (IBMX).

### Cell culture

All cell lines used in this study were mycoplasma negative. INS-1 832/3 cells were maintained in RPMI 1640 Media with GlutaMAX supplement (Thermo Fisher Scientific), 10% FBS, 10 mmol/l HEPES, 50 μmol/l 2-mercaptoethanol, 1 mmol/l sodium pyruvate and penicillin/streptomycin at 37°C in 5% CO_2_ [14]. Stably transfected cell lines overexpressing GPCRs were generated at AstraZeneca (Gothenburg, Sweden) or MedImmune (Cambridge, UK) using public-domain- or in-house-determined sequences for each receptor, with parental lines purchased from ATCC, ECACC or Invitrogen [15–17]. Overexpressing cell lines for experimental use were thawed from liquid nitrogen stocks into assay buffer on the day of the experiment for cAMP HTRF assays, and responses to agonist were assessed to confirm expected cell activity. A mirrorball system (TTP Labtech, Melbourn, UK) was used for the receptor ligand binding assay, described in detail in ESM Methods [18].

### Phage display selections

The bone marrow vault library (BMV_trp_), combined spleen library (CS_trp_) and DP47 library (DP47_trp_) were used for phage selections of single-chain variable fragments (ScFvs) that bound to the GLP1R. The first two rounds of selections, on 100 and 50 nmol/l soluble biotinylated human GLP1R ECD, respectively, were performed as previously described [16, 19]. For the third round of selection, cell surface selections were carried out on Chinese hamster ovary (CHO) cells overexpressing mouse GLP1R. Variable genes from antibodies of interest were cloned into pEU expression vectors for expression and purification of IgGs, as previously described [20].

#### cAMP HTRF accumulation assay

**Fig. 1:**
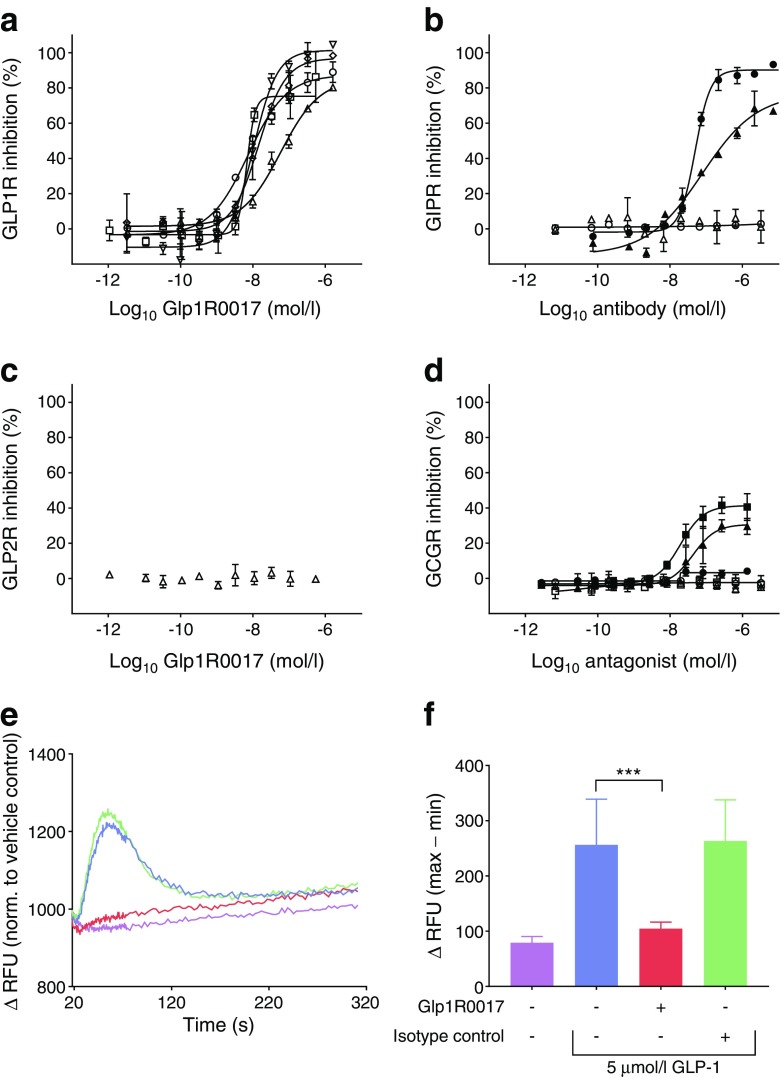
Glp1R0017 antagonises GLP-1-induced cAMP production in GLP1R-overexpressing cell lines, and has no effect on GIPR, GLP2R or glucagon receptor (GCGR) activity. Glp1R0017 was characterised for antagonism of GLP1R, GIPR, GLP2R and GCGR in a cell-based cAMP HTRF assay, and data were plotted using non-linear regression. (**a**) Antagonistic profiles of Glp1R0017 in CHO cells overexpressing mouse (circle), human (upright triangle), rat (square), cynomolgus monkey (inverted triangle) and dog (diamond) GLP1R. (**b**) Antagonistic profiles of Glp1R0017 (open symbols) and the antagonistic GIPR antibody Gipg013 (filled symbols) in CHO cells overexpressing human GIPR (triangles), and HEK293 cells overexpressing mouse GIPR (circles). (**c**) Antagonistic profile of Glp1R0017 in HEK293 cells overexpressing human GLP2R. (**d**) Antagonistic profiles of Glp1R0017 (white symbols) and the glucagon receptor antagonist des-His^1^-[Glu^9^]-glucagon (1–29) amide (black symbols) in CHO cells overexpressing human (triangles), mouse (circles) and rat (squares) GCGR. (**a**–**d**) Values have been normalised to the maximum activity of each receptor, defined by the total cellular cAMP production in the absence of peptide/IgG. Data are mean ± SE from duplicate wells, and the data shown are representative of at least three separate experiments. (**e**) Representative intracellular calcium responses of CHO human GLP1R cells to 5 μmol/l GLP-1 (blue), + 1 μmol/l Glp1R0017 (red trace), + 1 μmol/l R347 isotype control (green) or vehicle control (purple). Traces were normalised (norm.) to the vehicle control. (**f**) Mean intracellular calcium responses were measured in relative fluorescent units (RFU). Responses were calculated by subtracting basal fluorescence at 19 s from peak intensity. Data are mean ± SD, *n* = 16 wells (eight wells from each of two independent experiments). Statistical significance was assessed by one-way ANOVA with post hoc Bonferroni test. ***p < 0.001

Cell-based cAMP HTRF accumulation assays were used to screen Glp1R0017 for activity in overexpressing cell lines. Serial dilutions of the antibody and control peptides were prepared in assay buffer and plated using an ECHO525 acoustic liquid handler (Labcyte, Sunnyvale, CA, USA) to give an 11 point dose–response curve in duplicate. Cells were resuspended in assay buffer and then combined with serially diluted antibodies/peptides for 15 min of incubation at room temperature. An agonist challenge based on GLP-1 dose–response curves (see electronic supplementary material [ESM] Fig. 1) was added to samples using the ECHO550 acoustic liquid handler (Labcyte), and samples were centrifuged at 150 *g* for 1 min for mixing. Following 30 min of incubation at room temperature, cellular cAMP levels were measured using a cAMP dynamic 2 HTRF kit (Cisbio, Codolet, France) according to the manufacturer’s recommendations. After 1 h, plates were read on an EnVision plate reader (PerkinElmer, Waltham, MA, USA). This assay was also used for Schild analysis of Glp1R0017 in the CHO mGLP1R cell line. EC_50_ values were calculated using non-linear regression in GraphPad Prism (San Diego, CA, USA); dose ratios were determined and plotted to calculate dissociation constants.

#### Calcium measurements

CHO cells overexpressing human GLP1R were cultured in black poly-d-lysine-coated 384-well plates (15,000 cells/well; Greiner Bio-One, Stonehouse, UK) at 37°C overnight. Cells were washed with assay buffer, loaded with Fluo-4 NW containing 2.5 mmol/l probenecid (Thermo Fisher Scientific) for 30 min (37°C) and 15 min (room temperature), and then incubated with antibody for 15 min at room temperature before adding GLP-1. Fluorescence was recorded using FLIPR Tetra (Molecular Devices, Wokingham, UK) every 0.5 s for 1 min after agonist addition, followed by every 3 s for a further 4 min. Individual responses were normalised to vehicle control, and average responses were calculated by subtracting the basal fluorescence from the peak intensity. Statistical significance was assessed by one-way ANOVA with post hoc Bonferroni test.

#### cAMP FRET measurements

Single-cell measurements of cAMP were made using the fluorescence resonance energy transfer **(**FRET)-based sensor Epac2-camps [21], based on the method described for GLUTag cells [22]. INS-1 832/3 cells were seeded into 35 mm plastic dishes and, when 70–80% confluent, were transfected with 3 μg Epac2-camps DNA probe using 3 μl lipofectamine 2000 for 24 h. Cells were trypsinised and re-seeded onto Matrigel-coated 35 mm glass-bottomed dishes for experiments 24–48 h later. For each experiment, cells were washed with saline and then continuously perfused with saline ± test reagents. For the antibody experiment, cells were preincubated with 100 μmol/l Glp1R0017 diluted in saline for 15 min prior to the start of imaging.

Cells were visualised with a ×40 oil immersion objective on an inverted microscope (IX71; Olympus Southend on Sea, UK). A xenon arc lamp coupled to a monochromator (Cairn Research, Faversham, UK) controlled by MetaFluor software (Molecular Devices) was used to excite the cells at 435 nm (200–225 ms excitations) every 5 s. Cyan fluorescent protein (CFP) emission (470 nm) and yellow fluorescent protein (YFP) emission (535 nm) were monitored using an Optosplit II beam splitter (Cairn Research) and an Orca-ER digital camera (Hamamatsu, Welwyn Garden City, UK), and expressed as the CFP/YFP fluorescence ratio. A sliding average across 30 s was used, and responses were calculated by subtracting the maximum ratio at baseline from the maximum ratio during application of the test reagent. Changes in the CFP/YFP emission ratio were calculated as mean ± SEM, and statistical significance was assessed by one-way ANOVA with post hoc Bonferroni test.

#### Insulin secretion assays

Twenty-four hours before insulin secretion, INS-1 832/3 cells were seeded in 24-well plates at 5 × 10^5^ cells/well. Cells were washed in PBS and then incubated with or without Glp1R0017 in KRB containing 0.2% BSA for 1 h at 37°C in 5% CO_2_. Cells were then incubated for 2 h with test reagents in KRB. Supernatant fractions were collected and centrifuged to remove any cell debris. Insulin was measured in the supernatant fractions using a rat insulin assay (Meso Scale Discovery, Gaithersburg, MD, USA). Statistical significance was assessed by one-way ANOVA with post hoc Bonferroni test.

#### Animals

All animal care and experimental procedures were performed in accordance with the Animal (Scientific Procedures) Act 1986, local establishment usage guidelines and Animal Research: Reporting of In Vivo Experiments (ARRIVE) guidelines. The Project Licences authorising the work were approved by a local ethical review body (Animal Welfare and Ethical Review Body). C57/Bl6 mice were sourced from Charles River UK and group-housed in individually ventilated cages within a barrier unit with 12 h light/dark cycle and ad libitum access to chow diet and water. For the experiments, mice were randomised according to body weight.

#### Immunostaining

Pancreas tissue was fixed in 4% (wt/vol.) paraformaldehyde, dehydrated in 15% (wt/vol.) and 30% (wt/vol.) sucrose and frozen in optimal cutting temperature embedding media (VWR Chemicals, Radnor, PA, USA). Sections were cut (7–10 μm) using a cryostat and mounted directly on to Superfrost Plus glass slides (Thermo Fisher Scientific). Slides were incubated for 1 h in blocking solution containing 5% (vol./vol.) serum, 0.05% (vol./vol.) Tween-20 and 1% (wt/vol.) BSA, and then overnight in blocking solution with the primary antisera of interest against insulin, glucagon, GLP1R and tandem-RFP (ESM Table 1). Slides were washed with blocking solution and incubated with appropriate secondary antisera diluted to 1:300 for 1 h. Negative control slides were stained with secondary antisera alone. Cover slips were mounted using Hydromount (National Diagnostics, Atlanta, GA, USA) and DABCO before confocal microscopy (TCS SP8; Leica, Wetzlar, Germany).

Brain tissue was collected from mice perfused in situ with 4% (wt/vol.) paraformaldehyde and stored in 4% paraformaldehyde 30% (wt/vol.) sucrose prior to slicing at 25 μm. Sections were stained in suspension after sodium citrate antigen retrieval using protocols otherwise similar to those for pancreatic tissue, apart from the blocking solution, which did not contain any BSA.

#### Single-dose pharmacokinetics study

Two groups of six male C57/Bl6 mice (13 weeks old, mean weight 29.1 ± 0.2 g) were administered Glp1R0017 at 19.2 mg/kg either intraperitoneally or subcutaneously. A sparse sampling approach was used, collecting blood samples from animals across the groups at 0.5, 1, 2, 4 and 7 h after antibody dosing, and then every 24 h over a period of 5 days. At the 120 h endpoint, the animals were terminally anaesthetised using isoflurane inhalation, blood samples were collected via cardiac puncture, and death confirmed via cervical dislocation. Blood samples were collected into EDTA capillary tubes and centrifuged to obtain plasma. Quantitative analysis of plasma antibody concentration was performed in a Gyrolab assay according to the manufacturer’s guidelines (Gyros, Uppsala, Sweden), using a biotinylated idiotype antibody against IgG1 for capture (MedImmune, Cambridge, UK), and an Alexa-labelled sheep anti-human IgG for detection (The Binding Site, Birmingham, UK).

#### IPGTTs and OGTTs

GTTs were performed in 10-week-old male C57/Bl6 mice (mean weight: IPGTT 27.7 ± 0.2 g, OGTT 25.5 ± 0.3 g) after a 6 h fast, using *n* = 8 per group determined by power analysis where the effect size was 15%, and 80% power and significance <0.05. Depending on the group (detailed in Results), animals were dosed subcutaneously with antibody or saline 24 h prior to the GTT.

For the IPGTT, mice were dosed with 0.1 mg/kg liraglutide (Victoza; Novo Nordisk, Gatwick, UK) or vehicle subcutaneously 2 h before intraperitoneal glucose administration (2 g/kg). Blood glucose levels were determined from tail-prick blood samples using a hand-held glucometer (AlphaTrak; Zoetis, London, UK) at −120, 0, 15, 30, 45, 60, 90 and 120 min relative to the glucose challenge. For the OGTT, mice were orally gavaged with glucose (2 g/kg), and blood glucose levels were determined at 0, 15, 30, 45, 60, 90 and 120 min relative to the glucose challenge. At the end of the experiment, animals were euthanised using cervical dislocation. The AUC between 0 and 120 min was calculated, and statistical significance was assessed by one-way ANOVA with post hoc Bonferroni test.

## Results

### Selection of GLP1R antagonistic antibodies

Antibodies targeting the GLP1R were generated using naive phage display libraries in a series of selections on biotinylated human GLP1R ECDs, and cell surface selections on mouse GLP1R-overexpressing CHO cells. Following the third round of selection, clone diversity and enrichment was assessed by sequencing. Eighteen ScFv clones enriched in the cell surface selections were selected for functional analysis. The ability of ScFvs to antagonise GLP-1 stimulated cAMP production was assessed in a cell-based HTRF assay using mGLP1R-overexpressing CHO cells. Seven unique clones were identified as antagonistic, of which Glp1R0017 had the lowest IC_50_ value (238 nmol/l). On conversion into the IgG1 format, Glp1R0017 retained antagonistic activity and increased in potency (IC_50_ = 5.2 nmol/l; Fig. 1a).

### Glp1R0017 is a specific, competitive antagonist at the GLP1R across multiple species

Species cross-reactivity of Glp1R0017 was determined using a panel of CHO cells overexpressing mouse, rat, human, dog or cynomolgus monkey GLP1R in a cAMP-based HTRF assay. GLP-1 agonism was first assessed to determine an appropriate agonist challenge for each cell line (ESM Fig. 1). Glp1R0017 displayed antagonistic activity in each species assessed, and IC_50_ values were calculated (Fig. 1a, ESM Table 2). IC_50_ values ranged from 5.2 to 43 nmol/l, although these might have been affected by levels of GLP1R overexpression (ESM Methods and ESM Fig. 2). No Glp1R0017 agonist activity was observed at the mouse or human GLP1R (data not shown).

Specificity of Glp1R0017 for GLP1R was demonstrated with a cAMP assay using a panel of cell lines overexpressing other class B GPCRs. The antagonistic activity of Glp1R0017 was specific to GLP1R; no antagonistic activity was found in cells overexpressing the glucose-dependent insulinotropic peptide receptor (GIPR), glucagon like peptide-2 receptor (GLP2R) or glucagon receptor (Fig. 1b–d). In addition to cAMP responses, Glp1R0017 inhibited GLP-1-induced intracellular calcium responses in CHO cells overexpressing human GLP1R (Fig. 1e, f).

**Fig. 2:**
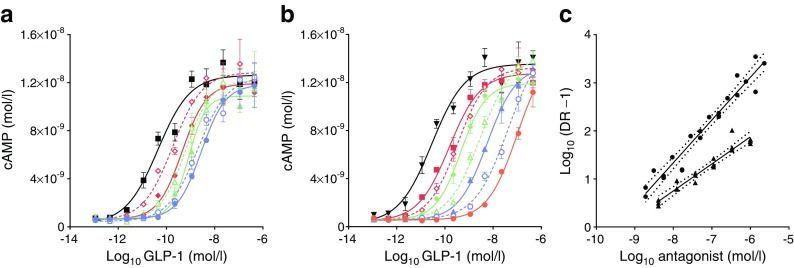
Glp1R0017 antagonism of the mouse GLP-1R is reversed through competition with GLP-1. (**a**) Dose–response curves of GLP-1 in the presence of Glp1R0017 at varying concentrations in CHO cells overexpressing mouse GLP1R. Concentrations of Glp1R0017 were: 4.1 nmol/l (dashed red line), 12.3 nmol/l (solid red line), 37 nmol/l (dashed green line), 111 nmol/l (solid green line), 333 nmol/l (dashed blue line) and 1 μmol/l (solid blue line). Vehicle group is shown in black. (**b**) Dose–response curves of GLP-1 in the presence of exendin 9–39 at varying concentrations in CHO cells overexpressing mouse GLP1R. Concentrations of exendin 9–39 were: 1.9 nmol/l (solid red line), 5.6 nmol/l (dashed red line), 16.8 nmol/l (solid green line), 50 nmol/l (dashed green line), 151 nmol/l (solid blue line), 453 nmol/l (dashed blue line) and 1.36 μmol/l (solid orange line). Vehicle group is shown in black. (**a**, **b**) Data are mean ± SE from duplicate wells; data are representative of at least three independent experiments. (**c**) Schild plot analysis of dose–response curves for Glp1R0017 (triangles) and exendin 9–39 (circles). Dose ratio (DR) is the ratio of the apparent GLP-1 EC_50_ in the presence of Glp1R0017 at a set concentration, over the GLP-1 EC_50_ in the absence of Glp1R0017. Points from three independent experiments are plotted, together with the 95% CI band for each line. The Schild plot *x*-axis intersect yields dissociation constant values of 466 pmol/l for Glp1R0017, and 315 pmol/l for exendin 9–39

The in vitro pharmacology of the antagonistic activity of Glp1R0017 on mouse GLP1R was investigated using Schild regression analysis, in which the effect of increasing concentrations of Glp1R0017 on GLP-1 dose–response curves was assessed. The maximum cAMP response to GLP-1 was not reduced by increasing Glp1R0017 concentrations, showing that the antagonistic effect of Glp1R0017 is not insurmountable (Fig. 2a). The same was observed with exendin 9–39 (Fig. 2b). The slope gradient of the Schild regression analysis for Glp1R0017 was 0.56, and the calculated dissociation constant was 466 pmol/l. Exendin 9–39 had a slope gradient of 0.89 and a calculated dissociation constant of 315 pmol/l (Fig. 2c).

### Glp1R0017 inhibits GLP1R function in INS-1 832/3 cells

Glp1R0017 was further characterised in INS-1 832/3 cells to show antagonistic activity on endogenous levels of GLP1R. Two aspects of GLP1R antagonism were assessed: antagonism of the cAMP response in live cell imaging, and antagonism of insulin secretion.

**Fig. 3:**
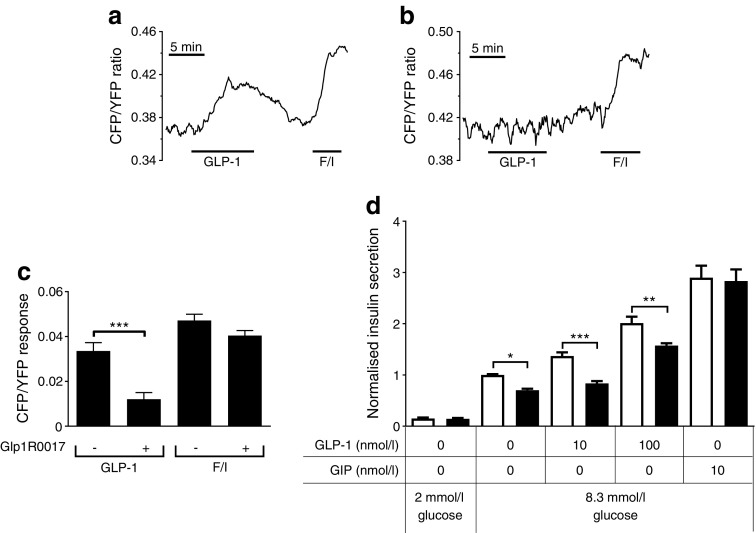
Glp1R0017 reduces GLP-1-stimulated cAMP and insulin secretion in the INS-1 832/3 cell line. (**a**) Example graph of changes in cAMP concentration in response to GLP-1 receptor activation. INS-1 832/3 cells transfected with Epac2-camps, to enable measurement of cAMP by FRET, were perfused with GLP-1 (100 nmol/l) followed by forskolin/IBMX (F/I; 10 μmol/l or 100 μmol/l), as indicated. (**b**) Example graph of changes in cAMP concentration following a 15 min preincubation with 1 μmol/l Glp1R0017. (**c**) Mean changes in the CFP/YFP emission ratio. Data are mean ± SEM; *n* = 19 for antibody-treated cells, and *n* = 26 for cells without antibody (monitored in 9–11 independent experiments per condition). Statistical significance was assessed by one-way ANOVA with post hoc Bonferroni test. ***p < 0.001. (**d**) Insulin secretion from INS-1 832/3 cells expressed relative to basal secretion in 8.3 mmol/l glucose measured in parallel on the same day. Cells were incubated with (black bars) and without (white bars) 100 nmol/l Glp1R0017. Data are mean ± SEM, *n* = 9–18 wells (from triplicates in 3–6 independent experiments). Statistical significance was assessed by one-way ANOVA with post hoc Bonferroni test. *p < 0.05, **p < 0.01, ***p < 0.001

To enable live cell measurement of cAMP, INS-1 832/3 cells were transfected with a FRET-based cAMP sensor, and the CFP/YFP ratio was recorded over time. A concentration of 100 nmol/l GLP-1 stimulated increases in cAMP (Fig. 3a), and this elevation in cAMP was reduced in cells preincubated with 1 μmol/l Glp1R0017 (Fig. 3b). Overall, preincubation of INS-1 832/3 cells with Glp1R0017 reduced the GLP-1-stimulated cAMP response by 64% (*p* < 0.001). In comparison, Glp1R0017 preincubation had no effect on forskolin/IBMX cAMP responses (Fig. 3c).

GLP1R activation and the subsequent increase in cAMP stimulated insulin secretion from pancreatic beta cells. Concentrations of 10 nmol/l GLP-1, 100 nmol/l GLP-1 and 10 nmol/l glucose-dependent insulinotropic peptide (GIP) stimulated insulin secretion, by 1.4-fold, 2-fold and 2.9-fold, respectively, in INS-1 832/3 cells. On incubation with Glp1R0017, 10 nmol/l GLP-1-stimulated insulin secretion was reduced from 1.4-fold to 0.8-fold (*p* < 0.01), whereas there was no significant effect on 10 nmol/l GIP-stimulated insulin secretion (Fig. 3d). This confirmed that Glp1R0017 specifically inhibited the function of GLP1R activation, with no off-target effects on the GIPR.

### Glp1R0017 binds and inhibits the function of GLP1R in vivo

**Fig. 4:**
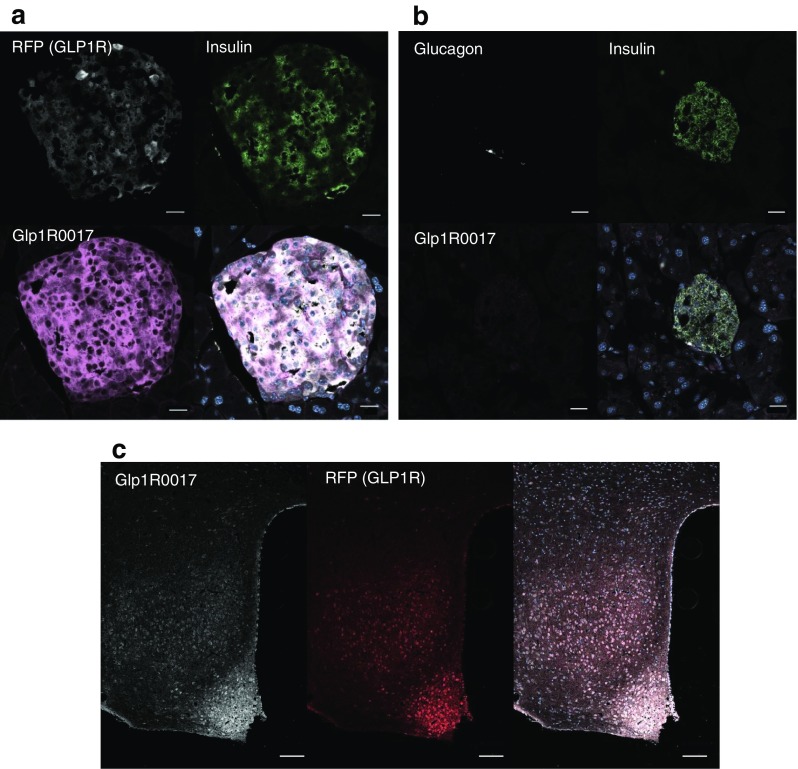
Glp1R0017 immunostains GLP1R within the Islets of Langerhans in mouse pancreas tissue. (**a**) Fixed pancreas sections from *Glp1r*-Cre/ROSA26-tdRFP mice were co-immunostained for RFP, insulin and GLP1R (using Glp1R0017). Nuclei were visualised with Hoechst stain. Scale bars, 20 μm. (**b**) Fixed pancreas sections from *Glp1r* ^−/−^ mice were co-immunostained for glucagon, insulin and GLP1R (using Glp1R0017). Scale bars, 20 μm. (**c**) Sections of 25 μm from the hypothalamus of *Glp1r*-Cre/ROSA26-tdRFP mice were co-immunostained for RFP and GLP1R using Glp1R0017. Nuclei were visualised with Hoechst stain. Scale bars, 100 μm

Following in vitro characterisation of Glp1R0017, pancreas tissue was collected from *Glp1r*-Cre/ROSA26-tdRFP mice [4] and *Glp1r* ^−/−^ mice [23]. The fixed and frozen tissue was used in immunostaining with Glp1R0017. In *Glp1r*-Cre/ROSA26-tdRFP tissue, Glp1R0017 bound to cells expressing the tandem red fluorescent protein (tdRFP) reporter in the islets of Langerhans and arcuate nucleus region (Fig. 4a, c). This immunostaining was depleted in control *Glp1r* ^−/−^ pancreas tissue, again showing that Glp1R0017 is specific for the GLP1R (Fig. 4b). This gave confidence that administration of Glp1R0017 to mice for functional studies would lead to GLP1R binding.

**Fig. 5:**
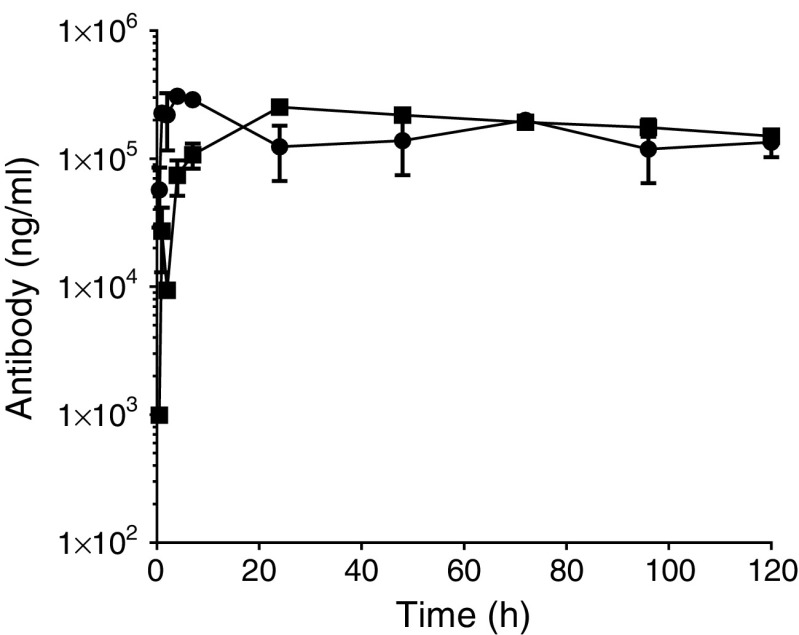
Glp1R0017 reaches C_max_ in 24 h when dosed subcutaneously, compared with 4 h when dosed intraperitoneally. Glp1R0017 was dosed at 19.2 mg/kg to two groups (*n* = 6) intraperitoneally (circles) and subcutaneously (squares), respectively. The concentration of antibody in plasma was then measured over 5 days (*n* = 3 at each time point). Three samples were taken per time point from 0.5 h to 96 h, with one sample missing at 0.5 h in the subcutaneous group and one at 72 h in the intraperitoneal group

Prior to functional studies in vivo, the pharmacokinetic properties of Glp1R0017 were assessed in C57/Bl6 mice over 5 days. A maximal concentration (C_max_) of 2.1 μmol/l was reached 4 h after 19.2 mg/kg intraperitoneal dosing, compared with a C_max_ of 1.7 μmol/l reached 24 h after 19.2 mg/kg subcutaneous dosing. At 120 h, the plasma concentration of Glp1R0017 was 0.9 μmol/l for the intraperitoneally dosed group, and 1 μmol/l for the subcutaneously dosed group (Fig. 5). Subcutaneous dosing was used for functional studies, 24 h prior to experiments.

**Fig. 6:**
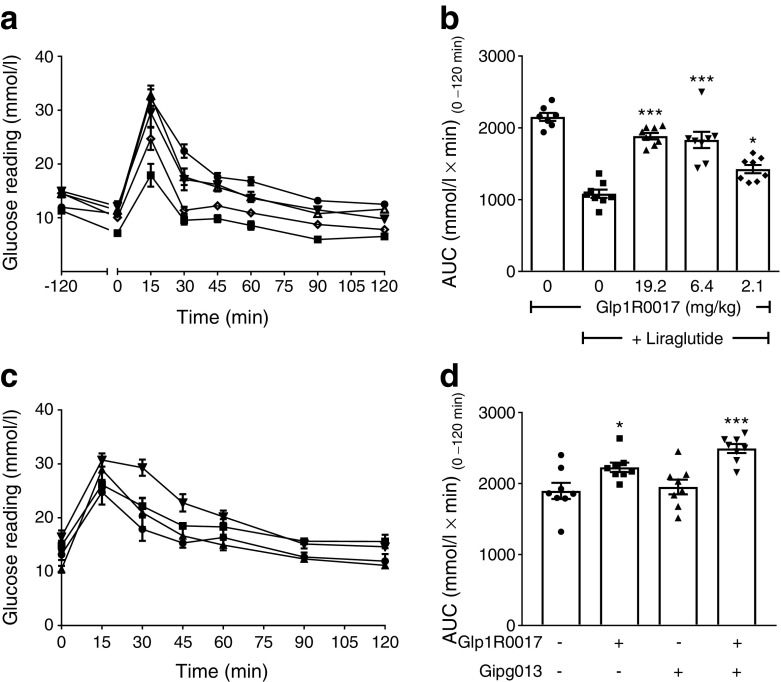
Glp1R0017 inhibits GLP1R in C57/Bl6 mice. (**a**) At 24 h after antibody dosing, and 2 h after liraglutide treatment (0.1 mg/kg), IPGTTs (2 g/kg) were performed. Vehicle only (circles), liraglutide only (squares) and liraglutide plus 19.2 mg/kg (upright triangles), 6.4 mg/kg (inverted triangles) and 3.1 mg/kg (diamonds) Glp1R0017. (**b**) AUC for glucose between 0 and 120 min. Data are mean ± SEM (*n* = 8). Comparisons were made with the liraglutide-only group, and statistical significance was assessed by one-way ANOVA with post hoc Bonferroni test. *p < 0.05, ***p < 0.001. (**c**) At 24 h after antibody dosing, OGTTs (2 g/kg) were performed. Circles, R347 isotype control 10 mg/kg; squares, Glp1R0017 9.6 mg/kg; upright triangles, Gipg013 100 mg/kg; inverted triangles, Glp1R0017 9.6 mg/kg with Gipg013 100 mg/kg. (**d**) AUC for glucose between 0 and 120 min. Data are mean ± SEM (*n* = 8). Comparisons were made with R347 group, and statistical significance was assessed by one-way ANOVA with post hoc Bonferroni test. *p < 0.05, ***p < 0.001

The ability of Glp1R0017 to block GLP1R in vivo was assessed by both IPGTT and OGTT in C57/Bl6 mice. An IPGTT was used to determine the antibody’s ability to inhibit the effect of the GLP-1 analogue liraglutide. Pre-treatment for 24 h with Glp1R0017 dose-dependently reversed the effect of liraglutide on glucose tolerance in IPGTTs (Fig. 6a, b). Glp1R0017 at 19.2 mg/kg completely abolished the effect of liraglutide on glucose tolerance, such that there was no significant difference in AUC compared with the vehicle-only group (Fig. 6b). This indicated that Glp1R0017 inhibits the GLP1R function in vivo.

To determine the ability of Glp1R0017 to block the endogenous incretin action of GLP-1, an OGTT was used. Glp1R0017 was dosed alone (9.6 mg/kg) or in combination with the GIPR antagonistic antibody Gipg013 (100 mg/kg) to block both the GIP and GLP-1 components of the incretin effect [16]. Gipg013 dosed alone did not have an effect on glucose tolerance. However, when Glp1R0017 was dosed alone, there was a small reduction in glucose tolerance compared with the isotype antibody control group (*p* < 0.05; Fig. 6c, d). When both GIPR- and GLP1R-mediated components of the incretin effect were blocked, glucose tolerance was further reduced (*p* < 0.001) compared with the isotype control antibody group (Fig. 6c, d). Hence, both antibodies are able to inhibit the action of endogenous incretin hormones on glucose-stimulated insulin secretion.

## Discussion

A monoclonal antagonistic antibody, Glp1R0017 targeting the GLP1R with nanomolar affinity has been generated using naive phage display. Schild regression analysis showed that Glp1R0017 antagonism of GLP1R is surmountable, as the maximal receptor activity was achieved with increasing competing concentrations of GLP-1. This suggests that Glp1R0017 is a competitive antagonist, although the slope of the Schild plots for Glp1R0017 and exendin 9-39 did not equal 1. This may suggest that equilibrium between antagonist and agonist had not been reached at the time of cell lysis within the cAMP assay, or that the inhibition was not a simple competition for the same binding site and the calculated dissociation constant should only be taken as an estimate.

Glp1R0017 not only inhibited cAMP production from the GLP1R, but also reduced the GLP-1-triggered increase in glucose-stimulated insulin secretion. Of interest, when assessing the effect of Glp1R0017 on insulin secretion from INS-1 832/3 cells, Glp1R0017 also significantly reduced insulin secretion in 8.3 mmol/l glucose without added GLP-1 (1–0.70-fold, *p* < 0.05). On further investigation, we found that GLP-1 was produced by INS-1 832/3 cells, increasing from 16.0 ± 1.6 pg/ml at 2 mmol/l glucose to 55.3 ± 3.0 pg/ml at 8.3 mmol/l glucose (*p* < 0.001, *n* = 9 wells) in the supernatant fraction, which probably explains the observed antibody effect in the absence of added GLP-1. Although GLP-1 secretion in the vicinity of INS-1 832/3 cells is hard to estimate, the observed inhibition suggests that the antibody can compete with very low effective GLP-1 concentrations in the picomolar range. In light of the endogenous GLP-1 secretion by INS-1 832/3 cells, this cell line could not be used to analyse antibody off-target effects on insulin secretion triggered by submaximal concentrations of GIP or other G_s_-coupled stimuli.

Immunostaining of the mouse pancreas with Glp1R0017 can be compared with that recently reported with 7F38A2 [24]. Both antibodies immunostained the beta cells of the islets of Langerhans in a GLP1R-dependent fashion. The Glp1R0017 cross-species reactivity, shown by the cAMP HTRF assay, suggests that immunostaining tissue from different species should be possible with Glp1R0017. This would complement immunostaining with MAb 3F52, which shows GLP1R localisation in monkey and human tissue [5]. MAb 3F52 has been characterised as an antagonistic antibody that directly blocks the GLP-1-binding site of human GLP1R [25]; characterisation of 7F38A2 antagonistic activity has, however, not yet been published. Glp1R0017 adds to these antibodies, providing an additional tool for studying GLP1R physiology in rodents and the other species (cynomolgus monkey, dog and human) with which it was cross-reactive. The specificity of Glp1R0017, demonstrated across a range of assays, provides a major benefit when compared with the peptide antagonist exendin 9-39, which has been shown to have off-target effects [12], although we cannot exclude potential interactions with unknown targets.

The in vivo ability of Glp1R0017 to inhibit GLP1R is a key finding of this study. Based on the pharmacokinetic study, it is estimated that the biological half-life of Glp1R0017 is longer than 120 h. Thus, with repeated doses, Glp1R0017 could be used to investigate chronic inhibition of GLP1R. The availability of both a GLP1R antagonistic antibody Glp1R0017, and a GIPR antagonistic antibody Gipg013 [16], enables investigation of the enteroinsular axis in vivo. The OGTT results are in line with GTT data from *Glp1r* ^−/−^ and *Gipr* ^−/−^ mice as, in both cases, only mild effects on glucose tolerance were observed in the individual knockout mice and single-antibody-treated animals [23, 26], whereas the combination of GLP1R and GIPR antibodies had a marked effect on glucose levels following the GTTs. These results support the idea that the two incretin hormones can compensate for each other, at least in part. A similar conclusion was reached in studies using single or double incretin receptor knockout mice [27]. Our finding that a single dose of Gipg013 did not significantly affect oral glucose tolerance, whereas Glp1R0017 caused a small but significant increase in plasma glucose levels after the OGTT, suggests that GLP-1 may be more important than GIP for the incretin effect in this mouse model, contrasting with studies using single and double incretin receptor knockout mice, which concluded that GIP was the more important incretin hormone [27].

The IPGTTs demonstrated that Glp1R0017 was able to block the effect of exogenously dosed liraglutide. This property of Glp1R0017 could be applied to the study of GLP-1 containing dual or triple agonists, which are emerging for the treatment of type 2 diabetes [28, 29]. These agonists are based on the concept that gastric bypass surgery, which commonly resolves the symptoms of type 2 diabetes, does not solely target one molecular pathway [30]. The balance of the different components within multi-agonists is important in development [14]; thus, Glp1R0017 may be useful for investigating the importance of the GLP-1 component within these agonists.

The ability of Glp1R0017 to block GLP1R in vivo opens up the possibility of studying extrapancreatic effects of GLP-1 in multiple species due to its cross-reactivity. There are a number of questions over how GLP-1 analogues exert the beneficial cardiovascular effects observed in clinical trials [8–10]. In summary, the GLP1R antagonistic antibody Glp1R0017, developed and extensively characterised here, provides a novel tool for investigating the GLP-1 component of unimolecular dual agonists for the treatment of type 2 diabetes, and for further understanding the physiology of GLP1R in vitro and in vivo. Glp1R0017 provides a new method to block GLP-1 receptors over several days in a range of species. It complements the use of *Glp1r* knockout mice by enabling transient and/or age-restricted GLP1R blockade, and may have advantages over exendin 9–39 when it is important to exclude potential cross-reactivity with related GPCRs.

## Electronic supplementary material

ESM (PDF 169 kb)

## Abbreviations

CFP: Cyan fluorescent protein
CHO: Chinese hamster ovary
C_max_: Maximal concentration
ECD: Extracellular domain
FRET: Fluorescence resonance energy transfer
GIP: Glucose-dependent insulinotropic peptide
GIPR: Glucose-dependent insulinotropic peptide receptor
GLP-1: Glucagon like peptide-1
GLP1R: Glucagon like peptide-1 receptor
GLP2R: Glucagon like peptide-2 receptor
GPCR: G-protein-coupled receptor
HTRF: Homogenous time-resolved fluorescence
IBMX: 3-isobutyl-1-methylxanthine
ScFv: Single-chain variable fragment
tdRFP: Tandem red fluorescent protein
YFP: Yellow fluorescent protein
