# Supplementary material for: Development and characterisation of a novel glucagon like peptide-1 receptor antibody
Source: Diabetologia. 2017 Nov 9;61(3):711–21. doi: 10.1007/s00125-017-4491-0 (PMC5890879; doi:10.1007/s00125-017-4491-0)
Supplement: Supplementary file 1 — (PDF 169 kb) [file 125_2017_4491_MOESM1_ESM.pdf]

Electronic supplementary material (ESM)

Methods

Receptor Ligand Binding Assay

A mirrorball system (TTP Labtech, Melbourn, UK) was used for the receptor ligand binding assay [1]. All reagents were prepared in HBSS containing 25 mmol/l HEPES and 0.1% (w/v) BSA. Serial dilutions of AlexaFluor647 tagged GLP-1 (Cambridge Research Biochemicals, Billingham, UK) +/- 0.5 µmol/l Exendin-4 were incubated with GLP1R overexpressing CHO cell lines (1000 cells per well), overnight at 4°C in the dark. Plates were read using the FL3 (650-690 nm) emission channel on the mirrorball, FL3 total readings (median fluorescence per event x count) were plotted.

Tables

ESM Table 1: Antibody details used for immunostaining

	Dilution	Source
dsRed	1/1000 – 1/500	Clontech, #632496
Insulin	1/100	Abcam, #7842
Proglucagon	1/100	Santa Cruz, #sc-7782

ESM Table 2: GLP-1 and Glp1R0017 activity in the GLP1R overexpressing CHO cell lines

	Mouse GLP1R	Human GLP1R	Rat GLP1R	Cyno GLP1R	Dog GLP1R
Glp1R0017 IC_{50} (mol/l)	5.21E-09	4.33E-08	5.3E-09	8.99E-09	11.7E-09
GLP-1 EC_{50} (mol/l)	3.92E-12	1.45E-12	4.51E-12	7.05E-13	6.07E-12

Mean IC_{50} values for Glp1R0017, and mean EC_{50} values for GLP-1 are tabulated.

Figures

ESM Fig 1: GLP-1 agonism in the GLP1R overexpressing CHO cell lines.

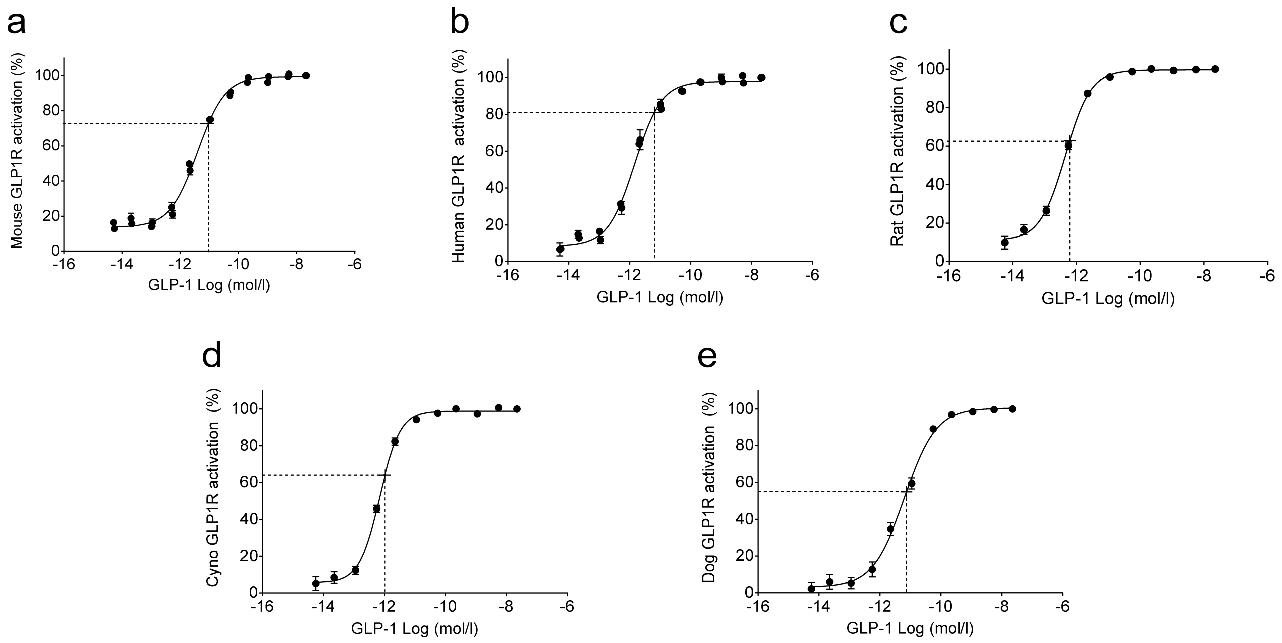

GLP-1 dose response curves for CHO cells overexpressing mouse GLP1R (A), human GLP1R (B), rat GLP1R (C), cyno GLP1R (D), and dog GLP1R (E) are displayed. The concentration of GLP-1 used as the agonist challenge in the antagonistic cAMP assays is marked on for each cell line.

ESM Fig 2: Binding of AlexaFluor647 tagged GLP-1 to the GLP1R overexpressing CHO cell lines.

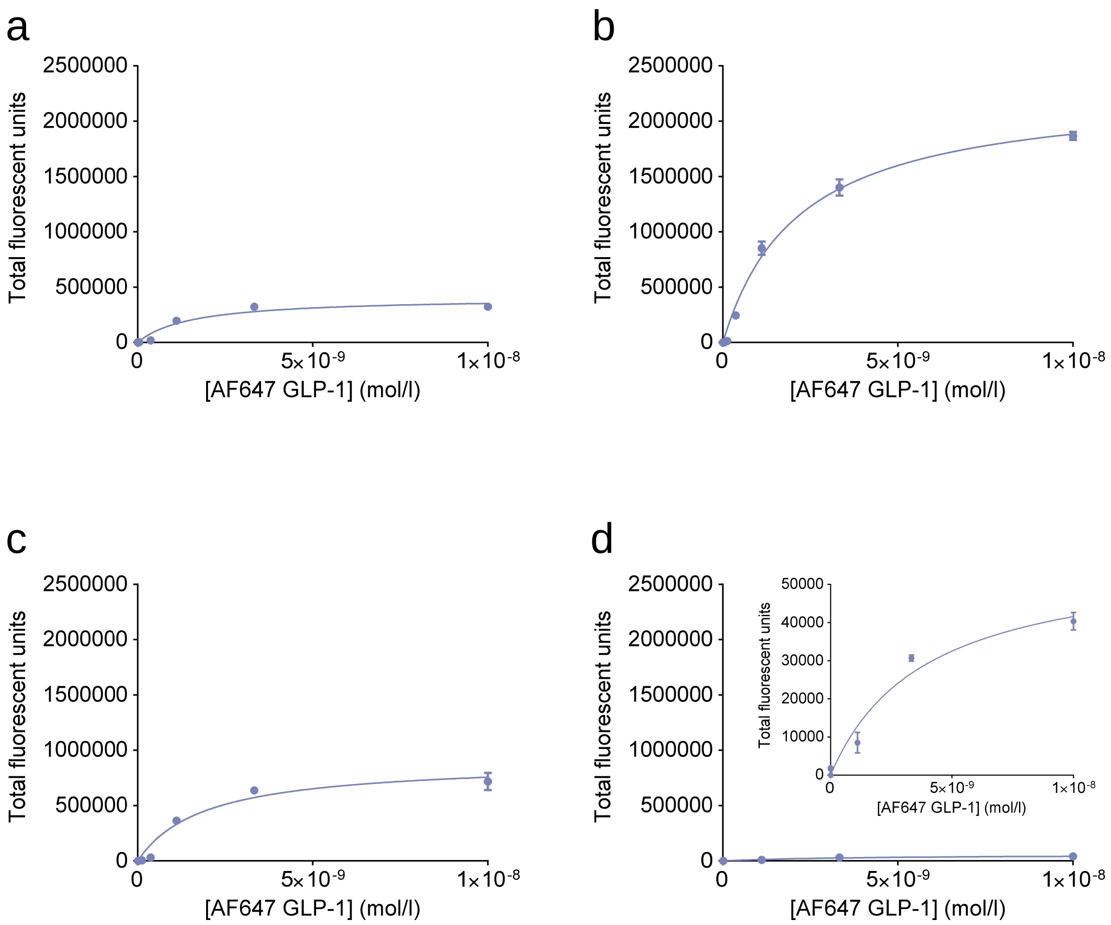

e

Cell Line	FL Max (RFU)		K_D (mol/l)	
	Mean	SD	Mean	SD
Human GLP1R	480000	170000	1.57E-09	8.42E-10
Rat GLP1R	1800000	690000	2.21E-09	5.71E-10
Cyno GLP1R	940000	77000	1.48E-09	4.46E-10
Mouse GLP1R	Below limits of detection			
Dog GLP1R	51000	7500	2.11E-09	1.54E-09

Representative AlexaFluor647 GLP-1 binding curves (n=3) are displayed for CHO cells overexpressing human GLP1R (A), rat GLP1R (B), cyno GLP1R (C), and dog GLP1R with zoom inset (D). The mean fluorescent max (FL max) and K_D for each cell line was calculated and tabulated (E). Non-linear binding curves were plotted using GraphPadPrism, with non-specific binding and background constrained to 0.

References

1. England E, Newton P, Neal F, Kitching L, Colley C and Rossant CJ (2015) Application of the mirrorball high-sensitivity cytometer to multiplexed assays for antibody drug discovery. J Biomol Screen 20: 536-544.
